# Do evolutionary life-history trade-offs influence prostate cancer risk? a review of population variation in testosterone levels and prostate cancer disparities

**DOI:** 10.1111/eva.12036

**Published:** 2012-12-11

**Authors:** Louis Calistro Alvarado

**Affiliations:** Department of Anthropology, University of New MexicoAlbuquerque, NM, USA

**Keywords:** challenge hypothesis, cross-cultural variation, male reproductive physiology, prostate cancer, testosterone

## Abstract

An accumulation of evidence suggests that increased exposure to androgens is associated with prostate cancer risk. The unrestricted energy budget that is typical of Western diets represents a novel departure from the conditions in which men's steroid physiology evolved and is capable of supporting distinctly elevated testosterone levels. Although nutritional constraints likely underlie divergent patterns of testosterone secretion between Westernized and non-Western men, considerable variability exists in men's testosterone levels and prostate cancer rates within Westernized populations. Here, I use evolutionary life history theory as a framework to examine prostate cancer risk. Life history theory posits trade-offs between investment in early reproduction and long-term survival. One corollary of life history theory is the ‘challenge hypothesis’, which predicts that males augment testosterone levels in response to intrasexual competition occurring within reproductive contexts. Understanding men's evolved steroid physiology may contribute toward understanding susceptibility to prostate cancer. Among well-nourished populations of Westerners, men's testosterone levels already represent an outlier of cross-cultural variation. I hypothesize that Westernized men in aggressive social environments, characterized by intense male–male competition, will further augment testosterone production aggravating prostate cancer risk.

## Introduction

**Figure 1:**
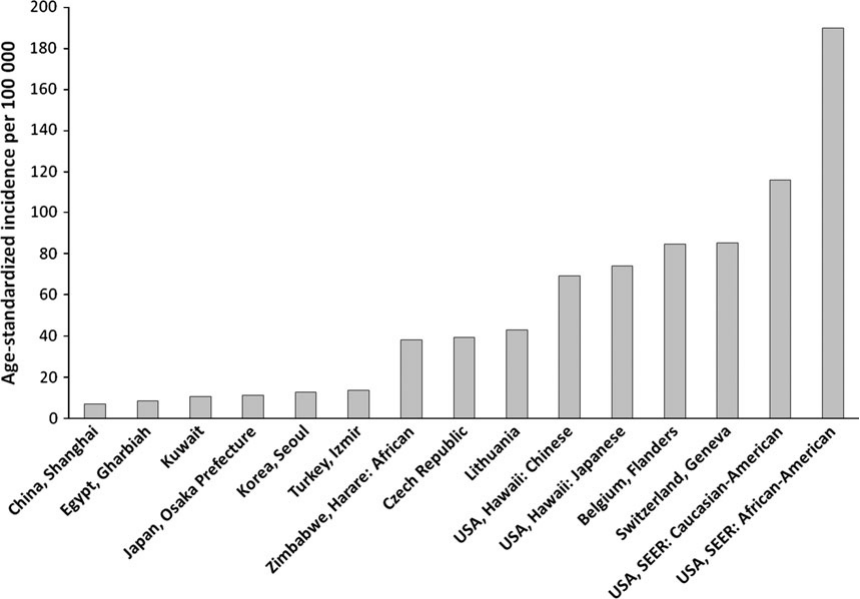
Age-standardized incident rates of prostate cancer. Data collected from Cancer Incidence in Five Continents, Vol. IX (Curado et al. 2007).

Prostate cancer represents a serious and rapidly growing health concern, and is now the second most common cancer among men. Western-industrialized societies have significantly higher rates of prostate cancer incidence, prevalence, and mortality than all others (Hsing et al. 2000; Kamangar et al. 2006). Global cancer statistics indicate that developed regions have a sixfold higher rate of prostate cancer incidence than developing regions (Kamangar et al. 2006). And for American men, prostate cancer is the second most common cancer and second leading cause of cancer death (Centers for Disease Control and Prevention and National Cancer Institute 2012), with an estimated 241,740 new cases and 28,170 deaths in 2012 (Howlader et al. 2012). Prostate cancer rates vary markedly both within and between populations (Fig. 1) (Hsing et al. 2000; Kamangar et al. 2006; Alvarado 2010). Understanding the underlying causes of this variation is important for cancer prevention and risk stratification.

Numerous risk factors for prostate cancer have been identified, including ancestry, diet, socioeconomic status (SES), and endogenous steroid concentrations (Grönberg 2003; Hsing and Chokkalingam 2006; Sanderson et al. 2006). However, these factors are frequently inconsistent, showing positive associations with prostate cancer in some studies and no, or even negative, associations in others (Meikle and Stanish 1982; Kolonel 2001; Sanderson et al. 2006; Roddam et al. 2008; Alvarado 2010). In this review, I focus on the effects of the steroid hormone testosterone, because substantial evidence suggests that cumulative, lifetime exposure to testosterone is a strong predictor of prostate cancer risk (Alvarado 2010). I argue that recognized risk factors for prostate cancer are likely proxies for chronically elevated testosterone exposure, and argue further that contradictory findings in the literature can be resolved by considering the specific ecological conditions that increase testosterone production. Evolutionary biologists have developed strong theories about how selective forces drive variation in testosterone levels between individuals, populations, and species (e.g., Wingfield et al. 1990; Ketterson and Nolan 1992; Bribiescas 1996, 2001a). Applying an evolutionary perspective to the problem of prostate cancer opens new avenues for understanding its etiology. It is my contention that commonly recognized risk factors, such as ancestry, dietary composition, and SES, will lead to higher prostate cancer rates only to the extent that they covary with socioecological factors influencing men's testosterone levels.

While other contributions to this Special Issue on Evolution and Cancer pertain to somatic evolution of neoplasia, this review addresses environmental and social conditions that affect cancer risk.

## Part 1: Elevated testosterone as a risk factor for prostate cancer

Androgens play a vital role in men's reproductive biology, initiating the process of sexual differentiation, supporting spermatogenesis, precipitating the development of secondary sexual characteristics, and maintaining sexual function in adulthood (Bribiescas 2001a; Krause 2006). Testosterone and its metabolites also influence the growth and functioning of the prostate gland, which secretes important components of seminal plasma (O'Malley 1971; Platz and Giovannucci 2004). Testosterone is transported to the prostate in circulation, where it is metabolized by the enzyme 5α-reductase into a more potent androgenic form dihydrotestosterone (DHT) (Matsumoto 2001). DHT binds to androgen receptors in the prostate with high affinity, promoting cellular proliferation of prostatic epithelium (Hsing 2001). Considerable evidence suggests that increased exposure to testosterone is associated with an elevated risk of sex-specific morbidity and mortality (Hamilton and Mestler 1969; Holden 1987), including prostate carcinoma (Henderson et al. 1982; Hsing et al. 2008; Alvarado 2010).

Animal models, clinical research, and *in vitro* studies have an established history of demonstrating the proliferative effects of testosterone on prostate cells and tumors (reviewed in Grönberg, 2003; Henderson et al. 1982; Hsing et al. 2008; Smith et al. 1994), though findings from epidemiological studies have been less consistent (Roddam et al. 2008; but see Shaneyfelt et al. 2000). Experimental studies in men, dogs, and rats showed that testosterone administration induced development of prostate cancer, while tumor size shrinks from androgen ablation treatment (Huggins and Hodges 1941; Pollard et al. 1982; Miyamoto et al. 2004). Similarly, a 7-year clinical trial of elderly men using finasteride, a drug that inhibits metabolization of testosterone to DHT, resulted in a 25% decrease in prostate cancer prevalence (Thompson et al. 2003). Furthermore, eunuchs who were castrated as young men do not develop prostate cancer, nor do men with hereditary deficiency of 5α-reductase (reviewed in Miyamoto et al. 2004). This is in contrast to habitual anabolic steroid users who show enlarged volume of central prostate tissue compared with age-matched, eugonadal controls (Jin et al. 1996).

**Figure 2:**
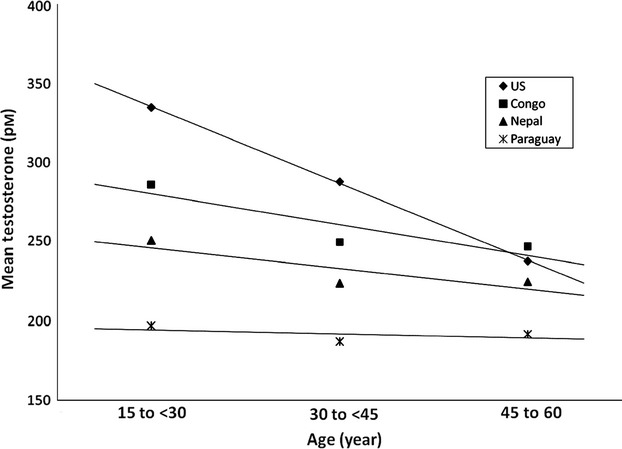
Mean testosterone levels across the lifespan of men from the USA and three preindustrial societies (Ellison et al. 2002).

On a larger cross-cultural scale, higher testosterone levels are found in Westernized men when compared with men from developing or traditional populations (Ahluwalia et al. 1981; Ellison et al. 1989, 2002; Christiansen 1991; Bentley et al. 1993; Bribiescas 1996, 2001a; Ellison and Panter-Brick 1996; Santner et al. 1998; Campbell et al. 2003; Kehinde et al. 2006), and the highest rates of prostate cancer are also found within developed regions of the world (Hsing et al. 2000; Kamangar et al. 2006; Curado et al. 2007). Population disparities in testosterone levels are most pronounced among young men (Fig. 2). Westernized men have higher testosterone levels but show a precipitous age decline in testosterone levels following early adulthood (Gray et al. 1991; Ellis and Nyborg 1992; Jankowska et al. 2000; Uchida et al. 2006), whereas men from preindustrial societies have lower testosterone levels followed by a less pronounced decline throughout the lifespan, and no appreciable difference exists between these groups in later life (Ellison et al. 1989, 2002; Bribiescas 2001a; Kehinde et al. 2006). Thus, prominent testosterone disparities in early life are not evident at later ages.

Despite accumulating evidence, the association between testosterone exposure and prostate cancer has proven controversial, largely because epidemiological studies have failed to find elevated testosterone levels in men with prostate cancer compared with healthy, age-matched controls (e.g., Andersson et al. 1993; Carter et al. 1995; de Jong et al. 1991; Hsing and Comstock 1993; Kubricht et al. 1999; Roddam et al. 2008; Sofikerim et al. 2007; but see Gann et al. 1996; Parsons et al. 2005; Shaneyfelt et al. 2000; Wolk et al. 1997). However, prostate cancer is primarily a disease of old age (Grönberg 2003; Hsing and Chokkalingam 2006), a period when men's testosterone profiles are diminished (Ellis and Nyborg 1992; Ellison et al. 2002; Gapstur et al. 2002; Kehinde et al. 2006). A major limitation of case–control studies of late-middle aged and elderly men is the lack of information on cumulative testosterone exposure over the life course.

Because the highest and most variable testosterone levels are found in young men, relative differences in men's cumulative hormone exposure can be most clearly identified during early adulthood (Grönberg 2003; Alvarado 2010), and these measures do correlate with prostate cancer risk. For example, African-American men have the highest incident rate of prostate cancer (Curado et al. 2007) and the highest average testosterone levels of all Western ethnic groups (Ross et al. 1986; Ellis and Nyborg 1992; Winters et al. 2001; Kehinde et al. 2006), but the latter difference is not detectable among older men (Ellis and Nyborg 1992; Kubricht et al. 1999; Pettaway 1999). Prostate cancer disparity between African- and Caucasian-Americans is evident as early as age 45, again suggesting that hormone exposure in young adulthood affects the trajectory of cancer risk in later life (Ross et al. 1986). Many studies have reported higher testosterone levels in young men from groups with higher prostate cancer incidences (Ross et al. 1986; Ellis and Nyborg 1992; Winters et al. 2001; Jakobsson et al. 2006; Kehinde et al. 2006). Moreover, meta-analyses that included only study samples of young men have reported that population differences in testosterone levels were positively and significantly associated with prostate cancer disparities in older men (Alvarado 2010, 2011).

Since men living in Westernized industrial conditions exhibit testosterone levels at an extreme for the observed range of human variation (Bribiescas 2001a), the question then becomes which factors result in further augmentation of testosterone production and whether these factors are associated with the prevalence of androgen-sensitive disease. Evidence for constitutional and ecological influences on steroid hormone production and metabolism is examined, with the prediction that socioecological factors supporting elevated testosterone will be associated with an increased risk of prostate cancer. Although this review primarily concentrates on variation in circulating testosterone levels, it is important to acknowledge that variation in androgen receptor sensitivity, carrier protein levels, and testosterone metabolization to DHT are all involved in androgenic action within the prostate (Hsing et al. 2008).

## Part 2: Life-history trade-offs affecting reproductive physiology

Life history theory provides a constructive theoretical framework for attaining greater insight in the evolutionary origins of a given trait. This becomes especially relevant if the trait under investigation is linked to a disease outcome that escalates with modernity, and may suggest an evolutionary mismatch in which a trait's adaptive function becomes maladaptive within an evolutionarily novel environment (Eaton et al. 2002). This section provides a brief overview of life history theory, while subsequent sections address how life history principles can inform our understanding of prostate carcinogenesis.

A primary determinant of evolutionary fitness is an organism's ability to capture energy from its environment and convert it into viable offspring, and a fundamental concept in life history theory is that energy allocated to one area is no longer available for investment in another. Because energy in the environment is finite, and because energy is not available once spent, selection favors strategic energy allocation across competing body systems in order maximize an organism's reproductive success. Differential energy allocations between growth, maintenance of soma, and reproduction define separate stages of the life course (Gadgil and Bossert 1970; Stearns 1989).

Mammals begin life dividing energy investment between growth and somatic maintenance (e.g., immune function), while reproduction is delayed. Once growth is nearly complete, the primary trade-off then shifts between reproduction and maintenance (Hill 1993; Kaplan et al. 2000). Investment in reproduction can be further divided into parental effort or mating effort. In mammals, females support the energetic cost of reproduction in the form of internal gestation and lactation (Clutton-Brock and Vincent 1991). Because females provide the greater minimum obligatory investment in offspring, females invest primarily in parental effort (Trivers 1972). Males, on the other hand, have more latitude in their reproductive decisions and are able to preferentially invest in either parental or mating effort (Ibid.), though direct male investment in progeny is quite rare among mammalian species (Clutton-Brock and Parker 1992). For males, spermatogenesis is energetically cheap, less than 1% of basal metabolic rate (Elia 1992), and male fecundity is relatively insensitive to energetic constraint (Bribiescas 2001a). In contrast, behavioral and secondary sexual characteristics are often costly for males in terms of energy expenditure, immunosuppression, or extrinsic mortality (Bribiescas 2001a; Muehlenbein and Bribiescas 2005; Ketterson and Nolan 1992; Redpath et al. 2006; Wilson and Daly 1985; Zahavi 1975). In vertebrates, testosterone has a critical role in modulating male life history and reproductive strategies (Bribescas, 2001; Ketterson and Nolan 1992; Wingfield et al. 1990) and will be discussed further in later sections.

## Part 3: Biosocial influences on testosterone production

Because several measures of endogenous testosterone are reported in the literature, a concise summary is presented in the Appendix for readers unfamiliar with these measurements.

### Nutritional status affects testosterone levels

**Figure 3:**
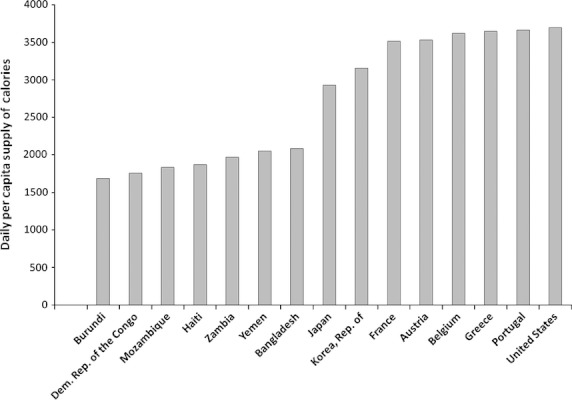
Daily per capita supply of calories, which represents the available calorie supply averaged across the population (United Nations Development Programme 2000).

Energy availability has a central role in calibrating men's testosterone levels (Bribiescas 2001a). And as nutritional status is especially variable between populations (Fig. 3), cross-cultural differences in energetic stress should have clear effects on interpopulation variation in testosterone levels (Ellison et al. 2002). In this section, between-population variation in men's testosterone levels is examined. Emphasis was placed on comparing Westernized populations with developing and small-scale societies, because shifts in dietary habits and activity patterns that accompany urbanization influence the expression of men's steroid physiology. Subsistence food production is often accompanied by chronic energy shortage, and men living under these conditions are generally incapable of meeting the energetic demands of physiological processes associated with elevated testosterone (Bribiescas 1996, 2001a; Ellison and Panter-Brick 1996; Ellison et al. 2002).

Bribiescas (1996, 2001a) proposed a functional explanation for observed variation in men's testosterone profiles. Males face a trade-off between somatic maintenance and reproduction that is modulated by testosterone's management of sexually dimorphic muscle mass. Although enhanced muscular development can improve competitiveness for mates, through male–male competition or female choice, it also raises metabolic rate and is energetically costly. According to Bribiescas, favorable energy availability promotes elevated testosterone to support muscular hypertrophy, while energetic stress results in down-regulated testosterone production and a diminished, thriftier phenotype. Whether diminution and augmentation of men's musculature is mediated through this specific pathway remains an interesting but tentative hypothesis, because some studies failed to substantiate that natural variation in men's testosterone levels exerts somatic consequences on muscle mass (Häkkinen and Pakarinen 1993; Ellison and Panter-Brick 1996; Campbell et al. 2003; Gettler et al. 2010) while other studies report only weak or indirect relationships (Campbell et al. 2007; Ellison and Panter-Brick 1996; Gettler et al. 2010; Lukas et al., #b^1002^).

It is well established, however, that men from populations under chronic energy shortage exhibit lower total and free testosterone levels in saliva and serum (Christiansen 1991; Bentley et al. 1993; Bribiescas 1996, 2001a; Ellison and Panter-Brick 1996; Campbell et al. 2003). Among Ariaal pastoralists of Northern Kenya, an examination of men's salivary testosterone levels and body composition in a sample of nomadic and settled Ariaal found that nomadic males had lower body fat percentage than settled males along with lower evening salivary testosterone values (Campbell et al. 2003). Nomadic men also showed a more pronounced age decline in both body fat and morning testosterone, which the authors attributed to energetic stress constraining testosterone levels of older men. Additionally, body fat was a positive predictor of morning testosterone levels, though testosterone levels were not associated with muscle mass. A subsequent study of the same population found that lean body mass was predicted by an interaction between evening salivary testosterone and androgen receptor sensitivity that was assessed using length of CAG repeats (Campbell et al. 2007). Conversely, an experimental examination reported dose-dependent effects of testosterone administration on young men's anabolic response without interaction from CAG repeat length (Woodhouse et al. 2003), and it remains uncertain to what extent the distribution of CAG repeats alters transcriptional activity of the androgen receptor gene.

Body fat percentage also positively predicted salivary testosterone levels in a sample of young Filipino men (Gettler et al. 2010). Once again, testosterone levels were not associated with muscle mass or strength measures, but there was an interaction between testosterone levels and sports participation in relation to lean body mass as well as grip strength. And in a study of men's reproductive hormones across urbanization gradient and economic strata within the developing population of South Africa, serum testosterone levels varied according to modernity and socioeconomic status (Gray et al. 2006a). Social groups within this study consisted of tribal men from rural villages, commercial farm workers, men residing in informal slum settlements (‘squatter camps’), urban inhabitants with access to public water and electricity, and urban professionals living in upper-class suburbs—with urban professionals representing the most Westernized lifestyle. Urban living and affluence were associated with both higher serum testosterone and body mass index, which is suggestive that increased testosterone production was supported by the superior energetic status of well-nourished urban professionals. Interestingly, the effect of nutritional constraint on testosterone secretion has been reported in comparisons of wild and captive chimpanzee males. Relative to their wild counterparts, captive chimpanzees are more sedentary with greater food availability, and captive males demonstrate higher urinary testosterone than wild males (Muller and Wrangham 2005). The comparison of captive and wild populations can provide a useful analog for domesticated animals. For example, household pets have less energetic constraint than would be expected in the wild. It seems reasonable to think that this ecological transition would have implications in regards to sex steroids levels and susceptibility to hormone-sensitive cancer. To my knowledge, no studies have compared steroid hormone levels in household pets and feral conspecifics. However, it is worth considering that aside from humans and synthetically induced cancer in laboratory rats, the only mammal that regularly develops prostate cancer is domestic non-neutered dogs, and sporadic reports of prostate cancer in cats have surfaced (Leroy and Northrup 2009).

In stark contrast to the marginal nutritional status of traditional or developing populations, Westernized men have an energy budget capable of supporting distinctly elevated levels of testosterone (Bribiescas 2001a). Indeed, it has been suggested that the near absence of severe energetic stress in Western populations permits male steroid physiology to operate at near maximal capacity (Bribiescas 2001b). Another potential constraint on hormone levels of non-Western men is greater exposure to pathogenic agents and infectious disease. Although it is overly simplistic to label testosterone as immunosuppressive, there is evidence that androgenic hormones can suppress some aspects of immune function, and conversely, immune challenges can suppress testosterone levels (reviewed in Muehlenbein and Bribiescas 2005; Muehlenbein 2008). If this is the case, then elevated testosterone would impose a disproportionate cost for men living in pathogen-rich ecologies of indigenous and developing habitats. Even in healthy US men, a mild immune challenge such as influenza vaccination can induce a subsequent decrease in salivary testosterone levels during the time of peak antibody production (Simmons and Roney 2009).

Under circumstances when Western men experience acute or chronic malnutrition, the subsequent physiological response is to lower testosterone levels (Klibanski et al. 1981; Lado-Abeal et al. 1999; Tomova and Kumanov 1999). Low levels of serum total testosterone and other signs of suppressed testicular function have been reported in young European men diagnosed with anorexia nervosa (Tomova and Kumanov 1999). Among collegiate wrestlers, lower serum testosterone levels were associated with low body fat as well as fat loss, and wrestlers showed decreased testosterone levels during the competitive season when energetic stress is most pronounced (Strauss et al. 1985). And obese men who have considerable energetic surplus in the form of abundant fat reserve still show lower serum testosterone after fasting (Klibanski et al. 1981). Moreover, a recent study found that a single missed meal in male college students can alter the male reproductive axis by decreasing luteinizing hormone and salivary testosterone levels (Trumble et al. 2010). But it is not entirely clear to what extent men's androgen production is constrained by transitory energy deficits; studies conducted within non-Westernized societies have found that seasonal workload and food shortage is not associated with lower salivary testosterone levels (Bentley et al. 1993; Ellison and Panter-Brick 1996). Similarly, a study of wild chimpanzees reported that males do not have lower urinary testosterone during the dry season when fruit is less available and energetic stress intensified (Muller and Wrangham 2005).

Although men's testosterone levels are affected by their nutritional status, the pattern diverges between Westerners and non-Westerners. For men living at subsistence level, higher adiposity can be indicative of superior energetic condition capable of supporting elevated testosterone (Ellison and Panter-Brick 1996; Campbell et al. 2003; Gettler et al. 2010). In contrast, high calorie consumption for Westerners, who are already well-nourished, is associated with fat accumulation and lower testosterone levels because of peripheral aromatization of androgens within adipose tissues (Kley et al. 1980, 1981; Pritchard et al. 1998). Large- and small-scale nutritional studies have found that measures of adiposity are negatively correlated with men's serum levels of total, free, and bioavailable testosterone in Westernized populations (Giagulli et al. 1994; Gapstur et al. 2002; Jensen et al. 2004; Tsai et al. 2006). And while greater energy consumption in Western men is correlated with higher adiposity and lower testosterone levels (Bishop et al. 1988; Pritchard et al. 1998), there is little empirical support showing a direct, causal relationship between diet composition and alterations in men's androgen production (Allen and Key 2000). An analysis of serum sex steroids levels in a sample of American monozygotic twins found that neither total nor free testosterone was associated with macronutrient consumption (Bishop et al. 1988; also see Field et al. 1994). Some androgenic metabolites were negatively correlated with calorie, fat, and protein consumption, while carbohydrate intake showed a positive correlation. However, the strongest determinants of men's testosterone was adiposity and body weight, both inversely related to testosterone levels, suggesting that dietary influences on testosterone levels were acting through the effects of body composition.

Nutritional analyses of Western men have not supported a direct effect of diet on testosterone levels after accounting for anthropometric differences (Key et al. 1990; Field et al. 1994; Allen and Key 2000). Allen and Key (2000) conducted a comprehensive survey of the literature on men's hormones, body composition, and diet among population samples that were drawn almost entirely from Westernized groups. They found that energy intake along with proportional intake of fat, protein, carbohydrate, and fiber as well as habitual diet (meat-eaters, vegetarians, and vegans) had a diminutive influence on hormone levels relative to the effects of body mass index and age. Some researchers have suggested that dietary habits do not adequately explain ethnic variation in testosterone levels of American men, because consumption of major nutrients does not differ significantly between American ethnic groups within the same social class while serum total and free testosterone levels do (Ross et al. 1986). Taken altogether, dietary composition among well-nourished Westerners does not appear to be a probable candidate for explaining variation in men's testosterone levels. Furthermore, variation in dietary regimens does not appear to contribute toward recognized differences in testosterone levels among Western ethnic groups. While dietary composition may not be associated with testosterone levels among Westernized men, this does not necessarily preclude other carcinogenic effects, outside of androgenic stimulation, of a Western-style diet on the prostate gland.

In summation, this section focused primarily on explaining cross-cultural variation in the testosterone levels of Westernized and non-Western men. Westernized men have greater energy availability and can support testosterone levels at the highest range of human variability, while men living at subsistence level are subjected to a higher degree of energetic stress and exhibit significantly lower testosterone levels. Variation in the dietary composition of Westerners does not seem to influence men's testosterone levels, although acute and chronic energetic stress does. Understanding the reproductive ecology underlying population differences in men's testosterone levels will go a long way in understanding prostate cancer disparities. This last point is particularly relevant because of evidence that population disparities in testosterone levels and prostate cancer are causally related (Alvarado 2010).

Until this point, I have focused on explaining between-population variation. The following sections will consider factors that affect testosterone levels and prostate cancer risk within populations. The first potential factor is ancestry.

### Ancestry is not associated with prostate cancer risk

Although ancestry is often thought to be a robust predictor of men's testosterone levels, an overview of this supposition reveals that supporting evidence is lacking. Ethnic variation in testosterone levels and prostate cancer rates has produced causal explanations for these disparities based on ancestry. Earlier it was mentioned that African-American men have the highest testosterone levels and greatest risk of prostate cancer (Ross et al. 1986; Ellis and Nyborg 1992; Winters et al. 2001), which have been attributed to a constitutional trait associated with African ancestry (Ellis and Nyborg 1992; Pettaway 1999). Contrary to this hypothesis, African-Americans have an extraordinarily high rate of prostate cancer when compared with African regions for which data are available, including West Africa (Curado et al. 2007; Kovi and Heshmat 1972; but see Odedina et al. 2006). Some researchers have taken the low incidence of prostate cancer found among African nationals as indirectly suggestive of lower testosterone levels in African populations (Ross et al. 1986), and there is empirical evidence that urban and indigenous groups of African men possess lower levels of serum total and free testosterone and salivary testosterone than either white or black American men (Ahluwalia et al. 1981; Ellison et al. 1989; Christiansen 1991; Bribiescas 2001a,b). Taken together, ancestry does not sufficiently explain elevated testosterone or prostate cancer risk in the ethnic group possessing the highest values. Furthermore, observed differences in the testosterone levels of young black and young white American men no longer exist after controlling for anthropometry and lifestyle factors (Rohrmann et al. 2007).

In contrast to higher testosterone levels of African-American men, lower levels of serum total, free, and bioavailable testosterone and lower salivary testosterone have been reported in men of Arabic and Asian descent: Chinese, Japanese, Kuwaiti, Omani, and Pakistani men (de Jong et al. 1991; Santner et al. 1998; Heald et al. 2003; Jakobsson et al. 2006; Kehinde et al. 2006), which again would appear to suggest that the expression of men's steroid physiology is a dispositional trait associated with ancestry (Ross et al. 1992; Jakobsson et al. 2006; Kehinde et al. 2006), but this position has also received little empirical support. One study reported that Chinese-American men have serum total testosterone levels that are significantly higher than Chinese nationals but not significantly different from white Americans (Santner et al. 1998). Similarly, Japanese-Americans exhibit much higher rates of prostate cancer than Japanese nationals (Severson et al. 1989; Shibata et al. 1997; Curado et al. 2007).

It is indisputable, however, that Arab and Asian men demonstrate remarkably low rates of prostate cancer (Yu et al. 1991; Kamangar et al. 2006; Curado et al. 2007), and cancer researchers have continued to search for risk factors associated with ancestry. Ross et al. (1992) reported that circulating levels of 5α-reduced metabolites were lower in Japanese men relative to other ethnic groups that exhibit higher risk of prostate cancer, which is intriguing since proliferation of prostatic epithelial cells is directed through testosterone metabolization to DHT by the enzyme 5α-reductase. Ross and colleagues proposed that a dispositional trait of Asian ancestry is responsible for a diminution of 5α-reductase activity, and that the lower prevalence of prostate cancer among Japanese men results from decreased exposure of prostate tissue to DHT. However, the indices of 5α-reductase activity used by Ross et al. (3α-androstanediol glucuronide and 3α-androsterone glucuronide) are strongly affected by adrenal steroid levels (Giagulli et al. 1989). Employing a more refined methodology that isolated gonadal sources of 5α-reduced metabolites, Santner et al. (1998) reported no notable differences in 5α-reductase activity between Caucasian-American and Chinese population samples; a similar result has since been replicated using a comparison of testosterone metabolism in white Australian and Chinese men (Jin et al. 2000). Accordingly, it seems that androgenic stimulation from higher testosterone levels, rather than 5α-reductase activity, is responsible for documented variation in prostate cancer rates between men of European and Asian descent (Kehinde et al. 2006). Some researchers have proposed that susceptibility to prostate cancer is determined by heightened sensitivity toward androgens resulting from the number of CAG and GGC microsatellites in exon 1 of the androgen receptor gene (Irvine et al. 1995; Giovannucci et al. 1997; Ross et al. 1998) and that the distribution of nucleotide polymorphisms is linked to ancestry (Kubricht et al. 1999; Pettaway 1999; Mohler 2007). This hypothesis has garnered limited support, because there are mixed reports of whether these genetic variants affect cancer outcomes (Grönberg 2003), and ethnic disparities in prostate cancer are not always consistent with ethnic variation in androgen receptor genotypes (Jin et al. 2000; but see Sartor et al. 1999). As such, ancestry does not effectively account for variation in testosterone levels and prostate cancer risk in ethnic groups possessing the lowest values, either.

In summation, ancestry does not adequately explain variation among ethnic groups with higher or lower testosterone levels, nor does it appear to explain variation among ethnic groups with high or low prostate cancer rates. This calls into question the efficacy of a disease model that is unable to predict either deleterious or protective effects.

### Socioeconomic status is often negatively associated with prostate cancer risk

Socioecological correlates of economic strata can influence male steroid production, as was apparent in the case of South African men discussed earlier. Fiscal resources are correlated with many aspects of health status (e.g., Smith et al. 1996a,b; Singh et al. 2003). Although national gross domestic product per capita is generally regarded as a positive predictor of health and longevity, this relationship more accurately captures relative differences between developing and developed countries (World Health Organization, 2002). Comparisons limited to affluent populations find that health outcomes are more closely tied to distribution of wealth within a nation's populace. Living in poverty within affluent countries is associated with a lower life expectancy, a greater prevalence of noncommunicable diseases, more prevalent drug and alcohol use, lack of access to healthcare, and higher rates of victimization from violent crime (Smith et al. 1996a,b; Ringel 1997; World Health Organization, 2002). Income disparities within industrialized nations often correlate with prostate cancer risk. Multiple studies have found increased morbidity and mortality from prostate cancer in men of lower social and economic status (Singh et al. 2003; Hall et al. 2005; Du et al. 2006 Sanderson et al. 2006; Rapiti et al. 2009). However, these findings were not replicated in a Norwegian population (Lund-Nilsen et al. 2000), which is of considerable interest and will be revisited.

## Part 4: The challenge hypothesis and prostate cancer risk

In this section, I draw on evolutionary theory to propose a new perspective on lifetime variation in testosterone exposure and consequent rates of prostate cancer. I build on research addressing between-population variation in testosterone levels, by further addressing social contexts that can lead to higher testosterone levels within Westernized populations—given that Westernized men can afford the energetic cost of elevated testosterone. More specifically, I propose that aggressive social environments place a premium on male–male competition and mating effort, formally termed the challenge hypothesis, which leads to chronically elevated testosterone and increased prostate cancer risk.

Wingfield et al. (1990) formulated the challenge hypothesis to explain variability in androgen production among seasonally breeding birds. At the beginning of the breeding season, males' testosterone levels raise from a nonbreeding baseline to a breeding baseline. Males then begin to establish territories, compete for mates, and intensify mate-guarding behavior as the breeding season progresses, exacerbating agonistic interactions with other males. During the period of mating competition, males' testosterone levels raise to a maximum baseline exceeding what is physiological necessary for male fecundity or secondary sexual characteristics. In monogamously mating bird species, mating effort decreases after a mateship is established as males move into a parenting role, and testosterone levels return to the breeding baseline from the physiological maximum. In contrast, polygynous male birds do not invest in parenting, compete for access to multiple females, and circulating testosterone does not decline from its physiological maximum during this time. Wingfield and colleagues presented convincing evidence that males' maximal rise in testosterone was elicited solely by aggressive challenges from male conspecifics in contexts directly relevant to reproduction. Thus, the effect of testosterone on aggressive competition complements its other effects on reproductive function and behavior. Elevated circulating testosterone is not without costs, however. In red grouse, for example, males treated with testosterone achieved higher mating success and were more likely to have multiple sexual partners than control males. And although treated males produced, on average, 2.5 more offspring than controls, they also suffered increased mortality from higher rates of predation (Redpath et al. 2006).

The same manner of challenges influencing testosterone secretion in birds can induce an analogous response in primate species. Direct male–male competition and aggression are associated with increased circulating testosterone. Furthermore, among group-living primates, males' testosterone levels are also responsive to the defense and maintenance of social status (Muller and Wrangham 2004; Archer 2006). The challenge hypothesis, adapted for primate species, has found support in prosimians (Cavigelli and Pereira 2000), monkeys (Beehner et al. 2006), apes (Muehlenbein et al. 2004; Muller and Wrangham 2004), and humans (Archer 2006). In chimpanzees, males that obtain sexual access to a noncontested mate do not show elevated testosterone levels, whereas males that must aggressively compete for access to females show a marked increase in testosterone. In a similar vein, an extensive meta-analysis found consistent support, across a wide range of studies, for the responsiveness of men's testosterone levels toward male–male competition (Archer 2006), and men with either more exposure to or involvement in violent behavior demonstrate higher testosterone levels (Dabbs and Morris 1990; Archer 1991, 2006; Dabbs et al. 1991; Banks and Dabbs 1996; Mazur 2006).

It seems reasonable that male hormonal response to challenges will have important health implications for men living in aggressive social environments, particularly those with the energetic resources to support high testosterone levels. For Westernized men from areas with a high intensity of male–male aggression and competition, the chronic nature of challenges may support chronically elevated levels of testosterone. Relative to other Americans, for example, African-Americans are subjected to a higher intensity of acute poverty, which produces downstream societal effects that amplify male–male competition, such that young African-American men experience more severe, repeated episodes of violence, as well as increased rates of homicide (Sampson and Wilson 1995; Geronimus et al. 1996; Ringel 1997). As would be expected in a social ecology with intensive male–male aggression, African-American men have repeatedly demonstrated serum total and free testosterone levels that are significantly higher than all other ethnic groups (Ross et al. 1986; Ellis and Nyborg 1992; Ettinger et al. 1997; Winters et al. 2001) and also have the highest incident rate of prostate cancer (Ghafoor et al. 2002; Merrill and Morris 2002; Curado et al. 2007; Altekruse et al. 2010). Irrespective of ethnicity, however, American men of low socioeconomic status experience increased rates of violent crimes (Ringel 1997), have higher serum testosterone levels (Dabbs and Morris 1990; Dabbs 1992), and suffer a higher rate of prostate cancer incidence and mortality (Singh et al. 2003; Hall et al. 2005; Du et al. 2006; Sanderson et al. 2006).

**Figure 4:**
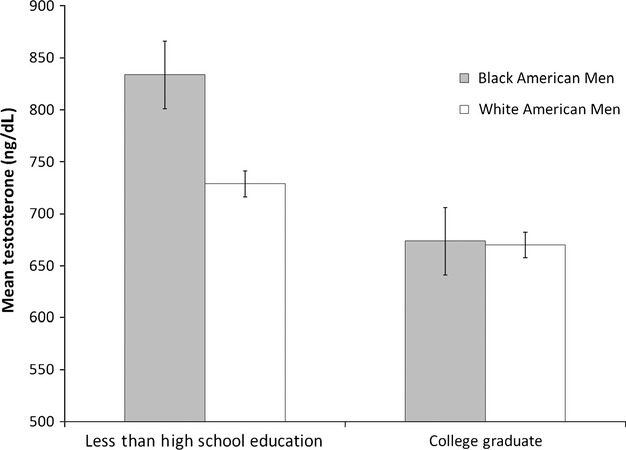
Testosterone levels according to ethnicity and education in a sample of male American veterans; median age of the sample was 37 years (redrawn from Mazur 1995).

Mazur (1995, 2006) argued that heightened dominance contests among economically disenfranchised men underlie recognized differences in testosterone levels between American ethnic groups. According to Mazur, impoverished conditions within American inner-city communities place a premium on dominance challenges between males, particularly young men. He argued that this association is evidenced by an overrepresentation of young, urban black men in the homicide statistics as both perpetrators and victims. Mazur analyzed ethnic variation in testosterone levels with respect to years of completed education using a large sample of US veterans, given that educated African-American men were less likely to come from impoverished urban areas. He found significantly different serum testosterone levels between young black and young white American men who had never completed high school, but this difference did not persist in college-educated men (Fig. 4). These data are suggestive that a social environment characterized by a high intensity of dominance contests can maintain elevated testosterone, as was evident among inner-city, African-American men.

It has long been observed that dominance contests, sometimes violent, occur more frequently among young men, leading researchers to term this phenomenon the ‘young male syndrome’ in which single young men are prone to risk taking and confrontational disputes to compete for potential mates (Wilson and Daly 1985). Aggression from male–male contests can often be explained in terms of investment in mating effort (e.g., Wilson and Daly 1985; Wingfield et al. 1990; Muller and Wrangham 2004; Archer 2006; Beehner et al. 2006). As reviewed in this section, the challenge hypothesis provides a practical framework to contextualize male–male competition and investment toward mating effort; the other integral component of this hypothesis addresses when males shift from mating effort to an investing parent.

Human males are especially unique in their extent of provisioning mates and offspring, and there is considerable evidence that this divergence from the reproductive strategies of other primates affected the course of human evolution (Lancaster and Lancaster 1983; Kaplan et al. 2000). As in any paternally investing species, human males face a particularly salient trade-off in reproduction between mating effort and parenting effort (Geary 2005). Numerous studies have examined hormonal changes occurring when single men transition to investing fathers (e.g., Gray et al. 2002; Muller et al. 2009; Gettler et al. 2011). Suppressed testosterone levels following the introduction of paternal care have been interpreted as a physiological shift away from mate-seeking behavior and toward investment in a current partner with shared offspring (Ibid.). Cross-sectional and longitudinal data have consistently reported that involved fathers demonstrate lower testosterone levels than unpaired males, and this finding is cross-culturally robust with the same general trend found in American, Canadian, Chinese, Filipino, Hadza, Jamaican, and Swahili men (Gettler et al. 2011; Fleming et al. 2002; Gray 2003; Gray et al. 2002, 2006b; Muller et al. 2009). Gettler et al. (2011) collected nearly 5 years of data on a nationally representative sample of Filipino men. They found that men with elevated levels of morning salivary testosterone were more likely to find mates and begin reproducing by the end of the observation period, which was then followed by an abrupt decline in testosterone levels after becoming fathers. These findings were particularly compelling, because longitudinal data showed that men's mating success was predicted by higher testosterone levels, while the demands of parenting effort down-regulated men's testosterone production.

Of course, considerable variation exists in the amount men invest in offspring, and men who have less involvement with their children deviate from the pattern described above. Muller et al. (2009) compared male parenting in two neighboring African populations. The Hadza and Datoga of Tanzania live in close proximity but have different modes of subsistence, leading to dissimilar patterns of pair-bonding and paternal involvement. Hadza live as traditional hunter-gatherers and are principally monogamous in which fathers are heavily involved with parental care. Datoga, on the other hand, subsist through pastoral farming and are patrilineal and polygynous, and males strive to acquire wives throughout their lifetime with little direct involvement in parenting. Thus, Hadza men shift investment from mating to parenting effort once a reproductive union is established, while Datoga men continue investing in mating effort after becoming fathers. These contrasting parenting strategies are manifested in the steroid physiology of Hadza and Datoga fathers. Hadza men show a pattern of higher salivary testosterone in single men and lower levels in pair-bonded fathers, while Datoga men maintained higher salivary testosterone after becoming fathers and throughout their reproductive years.

Some researchers have contemplated whether hormonal changes accompanying fatherhood affects health outcomes, including prostate cancer (Gettler et al. 2011). This would be especially relevant for Westernized men who experience a high degree of testosterone exposure. Since male parental involvement, rather than fatherhood in itself, appears to be the impetus for reduction of circulating testosterone (e.g., Gray 2003; Muller et al. 2009), it also becomes important to consider men's parenting strategies along with environmental circumstances that influence male investment patterns.

An obvious constraint to parenting effort is mortality risk, and there is evidence that local death rates affect the expression of reproductive strategies in which risky environments lead to preferential investment in mating effort (reviewed in Chisholm, #b^1001^). This may be the case for marginalized populations where men's ability to invest in children is compromised, such that parenting effort would be devalued and mating effort prioritized. In African-American communities, poverty is associated with significantly higher rates of intrinsic and extrinsic mortality (Geronimus 1996; Geronimus et al. 1999), and men from these communities are disenfranchised in additional ways that compromise paternal involvement. African-American men have disproportionate rates of incarceration, poverty, unemployment, and underemployment (Western 2002). From a life-history perspective, it is reasonable to expect males living in adverse and unpredictable conditions to invest preferentially in mating effort. Consistent with this logic, African-American communities display a suite of behavioral and physiological characteristics at the population level that are indicative of males prioritizing mating effort; men demonstrate a high degree of paternal disinvestment, male–male competition, and elevated testosterone levels. Put another way, widespread and severe poverty found among African-American communities is associated with higher rates of homicide and violent crime (Sampson and Wilson 1995; Geronimus et al. 1996; Ringel 1997), single mother households (Geronimus 1996; Mather 2010), elevated total and free serum testosterone (Ross et al. 1986; Ellis and Nyborg 1992; Mazur 1995, 2006), and might culminate into exceedingly high rates of prostate cancer (Du et al. 2006; Sanderson et al. 2006). Consequently, in addition to the immediate health concerns that impoverished living conditions pose, later deleterious effects may include persistently high testosterone and increased prostate cancer risk.

In summation, the literature reviewed in this section points to a direct relationship between male mating effort, aggressive challenges, and increased androgen production, a relationship that has been well documented across avian and primate taxa. Among Westernized men who have energetic resources to support the metabolic costs associated with elevated testosterone, there is evidence that being exposed to a higher frequency of aggressive challenges can result in chronically elevated testosterone levels. If living in an aggressive social environment contributes to prostate cancer disparities, this has important implications for prevention and risk stratification.

## Discussion

Modern Westernized environments represent a clear deviation from the environment in which male reproductive physiology evolved. Largely removed from energetic constraint and pathogen burden, Westernized men are capable of supporting distinctly elevated testosterone at the upper limit of human variability and amplifying the incidence of hormone-sensitive cancer. Variation in nutritional status can largely account for observed disparities in men's testosterone levels and prostate cancer between Westernized and non-Western populations, but not within Westernized populations—the populations at highest risk of prostate cancer. By incorporating a challenge hypothesis framework, another source of lifetime variation in testosterone exposure was proposed: Aggressive social environments affect prostate cancer incidence through the responsiveness of male androgen physiology to challenges, specifically among Westerners who are able to support the energetic costs of high testosterone levels. I reviewed literature which showed that ancestry, a widely recognized risk factor for prostate cancer, is in and of itself biologically unimportant when accounting for lifestyle factors. For instance, population disparities in testosterone levels of black-and white-American men become attenuated and nonsignificant when comparing among college-educated men from similar backgrounds (Mazur 1995, 2006). And in a nationally representative sample, there was no significant difference in testosterone levels of black-and white-American men after accounting for differences in anthropometry (age and body fat percentage) and lifestyle factors (drug use and physical activity) (Rohrmann et al. 2007). To reiterate, there is surprisingly little evidence to suggest that testosterone levels are a direct consequence of ancestry. And as discussed earlier, men of lower SES, regardless of ethnicity, demonstrate higher rates of male–male violence, higher testosterone levels, and higher prostate cancer. Using ancestry as a putative biomarker of prostate cancer risk is effective only to the extent which it tracks environmental circumstances and living conditions that influence cancer risk.

Additionally, I argued that poverty and compromised male investment lead to prioritized mating effort and increased male–male competition, culminating into chronically elevated testosterone and higher rates of prostate cancer. This general trend would be expected only if inequity in wealth distribution translated into more agonistic interactions between males at the population level. In other words, if the relationship between poverty and aggressive social environments is moderated, then there would be little expectation for lower SES to contribute to prostate cancer risk. Norwegian men, for example, deviate from the normally observed correlation between low SES and increased prostate cancer risk. This is particularly interesting because of the sizeable welfare program that is characteristic of Nordic social policy (Sachs 2006), which is associated with some of the lowest crime rates, violent or otherwise (Barclay et al. 2001). As such, Norway invests heavily in poverty reduction, boasts the lowest homicide rate within the developed world, and does not exhibit a concentration of prostate cancer among men of lower SES. Taken together, it would appear that comprehensive social programs might decouple socioeconomic differentials from male–male violence and prostate cancer risk, and may provide a surprising example of how improved social policies and poverty alleviation strategies are fundamental to the interest of public health.

And finally, the challenge hypothesis framework developed in this review may have occupational health implications, considering that men's testosterone levels vary according to occupational status (Dabbs 1992), and that some professions carry a disproportionate risk of prostate cancer (Demers et al. 1994; Zeegers et al. 2004). Dabbs (1992) and colleagues (1998) found that blue-collar workers have higher salivary and serum testosterone than white-collar workers. However, distinct social contexts within a profession can also give rise to differences in testosterone levels. Although lawyers as a group are white-collar workers, trial lawyers have significantly higher salivary testosterone than nontrial lawyers, which has been attributed to the polemical nature of face-to-face litigation (Dabbs et al. 1998). If this pattern of elevated testosterone from agonistic interactions persists across occupations, it seems reasonable to expect that men in professions with a higher intensity of competitive interaction would exhibit a greater incidence of prostate cancer. Findings from an extensive cohort study of 58,279 Western European men (ages 55–69 years) from 20 separate occupations are consistent with this reasoning (Zeegers et al. 2004). After accounting for individual characteristics and lifestyle factors (age, diet, drug and alcohol use, education, family disease history, and physical activity), it was police officers who showed the highest relative risk for prostate cancer. Indeed, prostate cancer risk increased 67% for each 10 years of occupational duty as a policeman. The framework proposed here can explain these seemingly peculiar associations between career choice and prostate cancer risk.

## Appendix: Testosterone Measures

The following overview will distinguish between different measurements of endogenous testosterone and will describe the functional significance of these measures. Men's serum total and free testosterone levels are frequently reported in the literature. Free steroid molecules are able to diffuse across cell membranes to interact with endocellular receptors and produce transcriptional activity that promotes androgenic signaling. However, free steroids are hydrophobic and must bind to carrier proteins for transport in peripheral circulation, such that only a small fraction of testosterone circulates in free form (Matsumoto 2001).

Serum testosterone is bound primarily to two carrier proteins, sex hormone binding globulin (SHBG) and albumin. Albumin binds loosely to testosterone, and albumin-bound testosterone is able to dissociate from its bond for entry into target tissues. SHBG, unlike albumin, binds testosterone with high affinity, rendering the molecule biologically inactive (Matsumoto 2001). Because total testosterone is a cumulative measure of diffusible and protein-bound testosterone, free testosterone is generally regarded as a more direct assessment of physiological availability (Matsumoto 2001; Rosner et al. 2007).

The literature also reports estimates of physiologically available testosterone, such as ‘bioavailable’ testosterone and free androgen index (FAI). Bioavailable testosterone is the combined portion of free and albumin-bound testosterone, whereas FAI is the quotient of total testosterone concentration/SHBG concentration (Rosner et al. 2007). Bioavailable testosterone correlates well with free testosterone (Vermeulen et al. 1999; Morley et al. 2002; Morris et al. 2004; Rosner et al. 2007), but FAI has shown poor correlations with free testosterone, particularly in men (Kapoor et al. 1993; Morris et al. 2004). Because of FAI's inconsistency, I relied on reports that used more dependable measures of serum testosterone: total, free, and bioavailable. In addition to serum, free testosterone levels can be collected from saliva (Ellison 1988; Lipson and Ellison 1989) as well as testosterone glucuronide excreted in urine and deconjugated for analysis (Muller and Wrangham 2004). Testosterone concentration in saliva is determined using radioimmunoassay procedures (Ellison 1988), and salivary testosterone levels are significantly correlated with serum-free testosterone levels (Vittek et al. 1985). Hormone analysis protocols used to assay salivary testosterone have also been adapted for urine (Muller and Wrangham 2004). These methods of noninvasive steroid hormone collection and analysis have proved invaluable for field research conducted outside of a laboratory setting.
